# To repair or to replace in mitral valve infective endocarditis? an updated meta-analysis

**DOI:** 10.1186/s13019-024-02767-y

**Published:** 2024-04-18

**Authors:** Ahmed K. Awad, Karim Wilson, Mahmoud A. Elnagar, Merihan A. Elbadawy, Mohamed H. Fathy

**Affiliations:** 1 Faculty of Medicine, Ain-Shams University, Cairo, Egypt; 2 Department of Cardiothoracic Surgery, Ain-Shams University Hospitals, Cairo, Egypt

**Keywords:** Infective endocarditis, Endocarditis, Mitral valve repair, Mitral replacement

## Abstract

**Background:**

Valve infective endocarditis (IE) is a potentially life-threatening condition that affects patients’ livelihoods. Current surgical options in mitral valve IE include mitral valve repair (MVr) or replacement (MVR). While each procedure boasts its merits, doubt remains as to which type of surgery is superior.

**Methods:**

We searched PubMed, Scopus, Web of Science, and Cochrane literature databases for studies comparing MVR and MVr in mitral valve IE. Any randomized controlled trial (RCT) or observational studies that compare MVR vs. MVr in mitral valve IE were eligible. Our dichotomous outcomes were extracted in the form of event and total, and risk and hazard ratio (RR)(HR) with 95% confidence interval (CI) and were pooled and calculated using RevMan 5.0.

**Results:**

Our study included 23 studies with a total population of 11,802 patients. Compared to MVR, MVr had statistically significant lower risks of both early mortality with RR [0.44; 95% CI, 0.38–0.51; *p* < 0.001] and long-term follow-up mortality with HR [0.70; 95% CI, 0.58–0.85; *p* = 0.0004]. Moreover, MVr was associated with a statistically significant lower risk of IE recurrence with RR [0.43; 95% CI, 0.32–0.58; *p* < 0.001]; however, no statistically significant differences between both groups in terms of re-operations with RR [0.83; 95% CI, 0.41–1.67; *p* = 0.60].

**Conclusion:**

Our results suggest that MVr was superior in terms of in-hospital mortality, long-term survival, and risk of recurrence without significance in valve reoperation. Therefore, MVr is appropriate as a primary treatment choice and should be considered whenever possible in most IE patients.

**Supplementary Information:**

The online version contains supplementary material available at 10.1186/s13019-024-02767-y.

## Key points

1. MVr showed superiority in terms of in-hospital mortality.
2. MVr has better long-term survival.
3. MVr has a lower risk of recurrence and valve reoperation.

**Supplementary Information:**

The online version contains supplementary material available at 10.1186/s13019-024-02767-y.

## Background

Valve infective endocarditis (IE) is a potentially life-threatening condition that affects patients’ livelihoods through either its occurrence or high risk of recurrence. Bacteria target the endocardium, particularly the heart valves, compromising their integrity [1]. This results in the development of vegetations that adhere to the valves causing incompetence or obstruction. These vegetations most frequently attack the mitral valve. Patients may not respond adequately to medical treatment which renders surgery their preferred method of treatment. Valve surgery may be absolutely essential to preserve heart function and to correct underlying damage caused by the disease [1].

Searching through the literature yields two established surgical treatments: mitral valve repair (MVr) and replacement (MVR). A plethora of scientific studies have been conducted on each method with varying results. While each procedure boasts its merits, doubt remains as to which type of surgery is superior. This meta-analysis, therefore, builds upon the previous meta-analysis [2] updating it with five new studies and tripling the patient pool. We aim to compare characteristics such as mortality, survival, recurrence, and reoperation of both procedures.

## Methods

The Preferred Reporting Items for Systematic Reviews and Meta-Analyses (PRISMA) [3] and Meta-analyses Of Observational Studies in Epidemiology (MOOSE) [4] guidelines were followed during the conduction of this systematic review and meta-analysis. Our study was registered with ID: 10.17605/OSF.IO/JUETP.

### Search strategy

Our search strategy was applied till February 2023 on several databases including PubMed, Scopus, Web of Science, and Cochrane literature databases to include studies comparing MVR and MVr. Any randomized controlled trial (RCT) or observational studies were eligible with no restrictions made regarding the language or publication period. Our keywords were mitral valve repair, annuloplasty, mitral valve repair, infective endocarditis, and mitral endocarditis. Moreover, the included articles’ references were searched manually for any relevant or missed eligible studies. We excluded Reviews, letters to editors, conference abstracts, and studies that investigate either operation solely, studies that include IE and non-IE mitral valves, studies that included patients with congenital heart diseases or don’t satisfy the modified Duke criteria to diagnose IE.

### Eligibility criteria

Studies that investigate the mitral valve surgery repair versus replacement for mitral valve IE, published in peer-review journals reporting separate outcomes data for the two surgical groups were included. Our PICO (Patients, intervention, control, and outcome) was P; patients with mitral valve infective endocarditis, I; mitral valve repair/annuloplasty, C; mitral valve replacement, O; primary outcomes: early in-hospital mortality, long-term follow-up mortality, while secondary outcomes: risk of IE recurrence, and risk of reoperations.

### Data extraction and quality assessment

Both two independent authors (M.H.F, M.E) applied our eligibility criteria through first title and abstract screening and then full-text screening to select eligible articles. Any disagreement was solved by a third author (A.K.A). Articles had to have a baseline characteristics table for their patients with at least one of our primary or secondary outcomes. Baseline characteristics were extracted by two independent authors (K.W, M.H.F) along with mortality, survival, recurrent endocarditis, and valve reoperation as outcomes. The risk of bias was assessed using the Cochrane ROBINS-I tool [5] for observational studies. Two independent reviewers (A.K.A and M.A.A) screened the methodological quality of included studies and any discrepancies were resolved by a senior author (M.H.F).

### Data analysis

The inverse variance method was used to pool study estimates, and the restricted maximum-likelihood estimator was used to estimate between-study heterogeneity. The risk ratio (RR) or Hazard ratio (HR) with a 95% confidence interval (CI) are provided as effect size estimates. Random-effects meta-analysis models were utilized to investigate whether the results were sensitive to model choice. Forest plots were drawn, and the shaded boxes represent the point estimate for each individual trial, and the horizontal line extending from each box represents the upper and lower limits of the 95% CI. The diamonds represent the overall effect size. Meta-regression was used to explore the effects of mean age, gender, diabetes mellitus (DM), hypertension (HTN), and left ventricular ejection fraction (LVEF) on our outcomes. All analyses were performed using RevMan 5.0.2. and R software (version 4.2.0) [6, 7].

## Results

### Study selection

Our search strategy retrieved a total of 1817 studies. Following the title and abstract screening and removing the duplicates, 40 full-text articles were evaluated for eligibility. Following the full-text screening, 23 observational studies [8–30] met our criteria and were included in our meta-analysis. Further details of the PRISMA flow chart can be found in Supplementary Fig. 1 and Table 1.

**Table 1:** Baseline characteristics of included studies

Study	Country	Study type	Total sample size (n)		Mean follow up time (years)		Age (mean(SD))		Sex (female) n (%)		DM n (%)		HTN n (%)		AF n (%)		LVEF (mean (SD))	
			MVr	MVR	MVr	MVR	MVr	MVR	MVr	MVR	MVr	MVR	MVr	MVR	MVr	MVR	MVr	MVR
Scheggi 2019	Italy	RC	33	57	3	3	63.7(13.1)	63.6 (13.9)	6(18)	20(35)	4(12)	16 (28)	13 (39)	33 (58)	NA	NA	58.5 (9)	60(10.5)
rutman 2005	Austria	RC	34	34	3.14	3.70	51.5(17)	53.2(13.1)	12(35.2)	17(50)	6 (17.6)	6 (17.6)	NA	NA	NA	NA	49 (12)	53(12)
Miura 2014	Japan	RC	36	21	5.3	5.3	57(17)	55(17)	27 (75 )	8 (38 )	6 (17 )	2 (10 )	NA	NA	2 (6 )	2 (10 )	NA	NA
Lee 2018	China	RC	38	33	3.5	3.5	42.3(18.8)	53.7(15.6)	14 (36.8)	13 (39.4)	4 (10.5)	11 (33.3)	NA	NA	NA	NA	70.2(6.8)	69.8(14.5)
Jung 2011	Korea	RC	41	61	4.7	4.7	34.4(16.9)	43.1(14.9)	22(53.6)	28(45.9)	5 (12.2)	8 (13.1)	4 (9.8)	9 (14.8)	NA	NA	64.1(4.2)	62.7(6.2)
Defauw 2020	Netherlands	RC	97	52	5.5	5.5	57 (13)	61 (13)	77(79.3)	30(57.7)	8 (7.9)	13 (25.0)	35 (36.1)	20 (38.4)	18 (18.6)	5 (9.6)	NA	NA
cuerpo 2019	Spain	RC	68	301	1	1	61 (46–70)	63 (53–73)	16(23.5)	90(29.9)	NA	NA	28 (41.2)	148 (49.2)	6 (8.8)	56 (18.6)	NA	NA
helmers 2021	USA	RC	47	47	6.2	6.2	51.3 (13.6)	52.6 (18.0)	16 (34.0)	23 (48.9)	6 (12.8)	7 (14.9)	21 (44.7)	28 (59.6)	NA	NA	58.0 (11.6)	59.3 (12.0)
shang2009	USA	RC	56	33	NA	NA	NA	NA	30(33.7)	NA	17	12	NA	NA	NA	NA	NA	NA
solari 2019	Belgium	RC	155	37	10.20	12.19	60.1 (13.8)	64.6 (11.5)	46(29.7)	19(51.4)	33 (21)	7 (18.9)	NA	NA	NA	NA	NA	NA
tepsuwan 2019	Thailand	RC	38	76	2.76	3.40	44.1 (15.8)	47.5 (15.0)	17(44.7)	21(25)	11	10	NA	NA	NA	NA	58.5 (9)	60(10.5)
tomisc 2018	Netherlands	RC	51	32	3.7	3.7	55 (14)	60 (13)	13 (26)	15 (47)	6 (12)	8 (25)	16 (31)	11 (34)	6 (12)	2 (6)	NA	NA
toyoda2017	USA	RC	367	1603	6.6	6.6	54.9 (15.3)	57.4 (14.6)	124(33.8)	703(43.9)	31 (8.4)	203 (12.7)	175 (47.7)	881 (55.0)	85 (23.2)	405 (25.3)	NA	NA
wang2014	New Zealand	RC	25	35	3.9	3.9	43.1 (16.3)	52.1 (15.7)	11(44)	14(40)	2 (8.0)	3 (8.6)	6 (24.0)	12 (34.3)	2 (8.0)	17 (48.6)	58.0 (11.6)	59.3 (12.0)
Wilhelm 2004	Switzerland	RC	57	97	7	7	NA	NA	NA	NA	NA	NA	NA	NA	NA	NA	NA	NA
Mihaljevic 2004	USA	RC	21	32	4	4	NA	NA	NA	NA	NA	NA	NA	NA	NA	NA	NA	NA
Sternik 2002	USA	RC	16	28	3.25	3.25	NA	NA	NA	NA	NA	NA	NA	NA	NA	NA	NA	NA
yamaguchi 2006	Japan	RC	14	7	NA	NA	55.5 (12)	61 (11)	4(28.6)	4(57.1)	NA	NA	NA	NA	NA	NA	NA	NA
oliver 2022	France	PC	115	37	2.33	2.33	59 (15)	58 (14)	37(32.2)	12(32.4)	15 (13)	9 (24)	NA	NA	13 (11.3)	2 (5.4)	NA	NA
brescia 2021	USA	RC	181	79	3.7	2.7	54 (15)	54 (14)	53 (29)	35 (44)	28 (15)	25 (32)	71 (39)	46 (58)	NA	NA	56 ( 11)	61 (9)
Musci 2010	Germany	RC	32	17	3.59	3.59	44.3 (11)	44.3 (11)	20	10	NA	NA	NA	NA	NA	NA	NA	NA
Gammie 2005	USA	RC	1965	4662	9	9	56	67	NA	NA	NA	NA	NA	NA	NA	NA	NA	NA
LEE 2024	Taiwan	RC	467	467	NA	NA	NA	NA	NA	NA	NA	NA	NA	NA	NA	NA	NA	NA

RC; retrospective cohort, PC; prospective cohort, MVr; mitral valve repair, MVR; mitral valve replacement. DM; diabetes milletus, HTN; hypertension, AF; atrial fibrillation, LVEF; left ventricular ejection fraction; SD; standard deviation; N; number of patients, NA; not available

### Summary of the included studies

All our studies were observational studies with a total population of 11,802 patients, with 7,848 and 3,954 patients underwent MVR and MVr, respectively. Most of our patients were males with 84% in MVr and 86% in MVR group with a mean age of 55.2 (13.7) and 57.8 (14.2) in MVr and MVR groups, respectively. The most common indication for surgery was mobile vegetation in both MVr (46.3%) and MVR (42.7%) groups. Further details about our baseline can be found in Table 1.

### Risk of bias

Applying ROBINS-I tool to our observational studies, 20 reports have low risk of bias quality, while three have moderate risk of bias; two due to bias in the measurement of outcomes and one due to missing data. Further details of quality assessment can be found in Supplementary Table 2.

### Outcomes

**Fig. 1:**
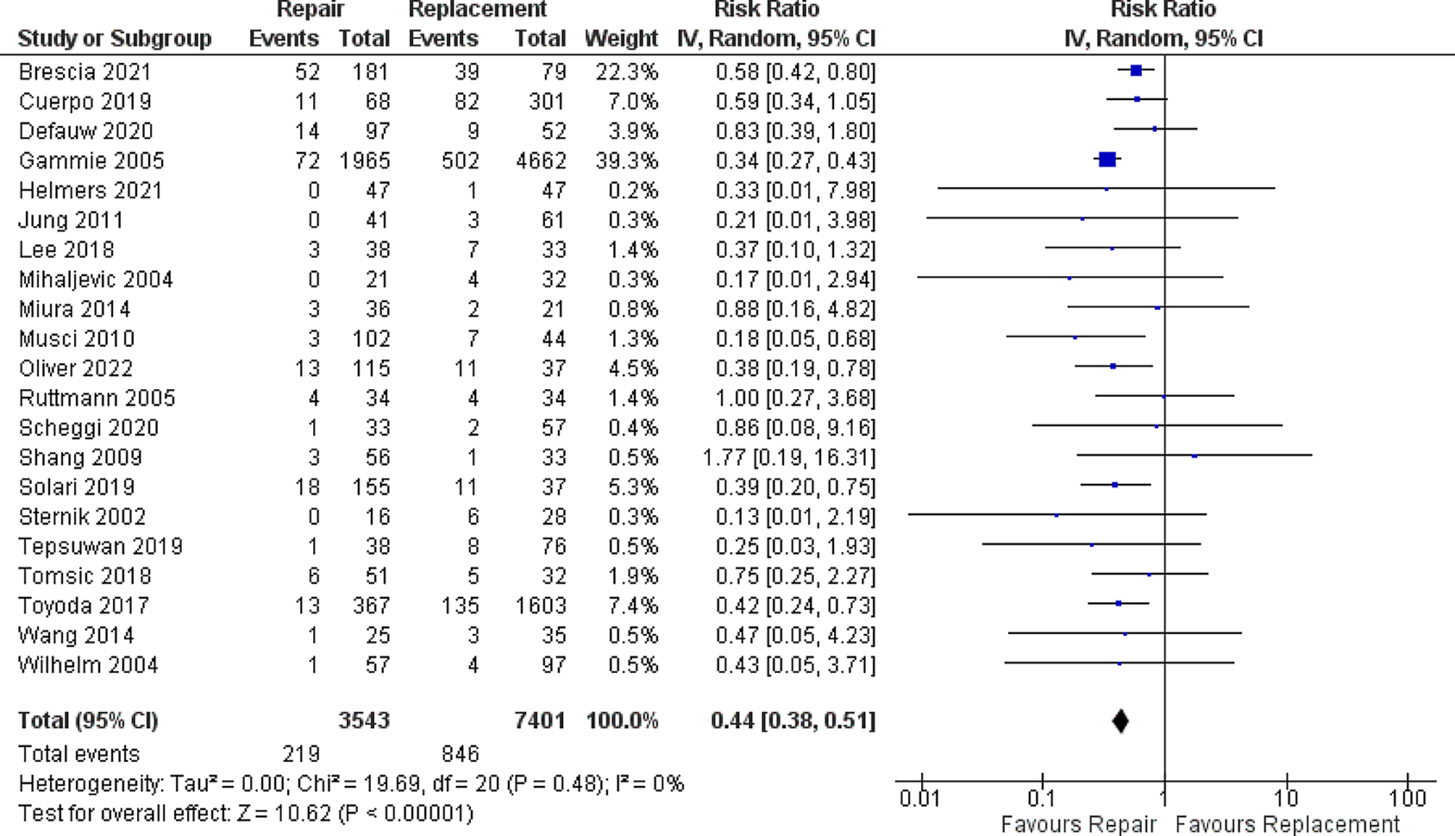
Forrest plot analysis of early mortality

**Fig. 2:**
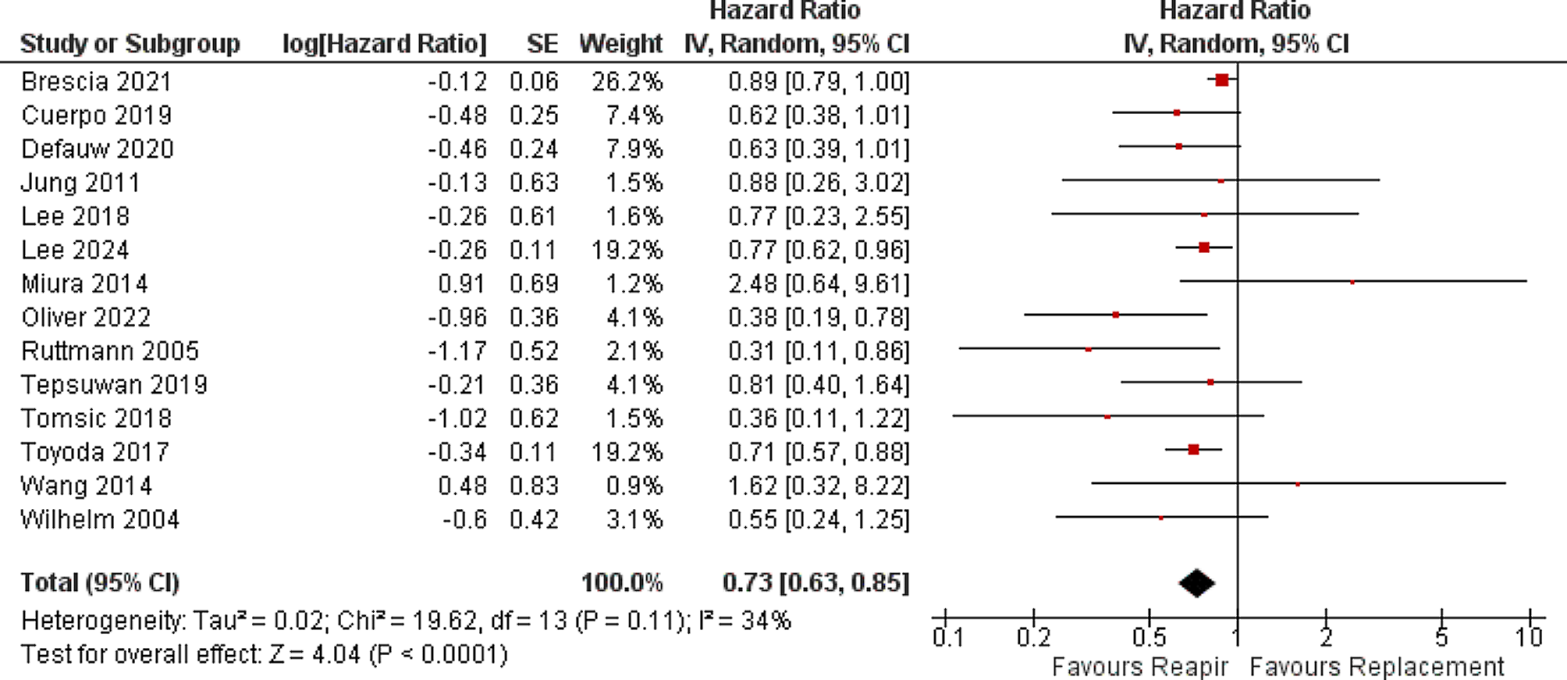
Forrest plot analysis of late mortality

In terms of early mortality, the analysis of 21 studies with 3543 patients in the MVR group and 7401 patients in the MVr group showed that patients who underwent MVr had a statistically significant lower risk of early in-hospital mortality with RR [0.44; 95% CI, 0.38–0.51; *p* < 0.001] **(**Fig. 1**).** No heterogenicity was observed [*p* = 0.48, I^2^ = 0%]. Moreover, in the analysis of 14 studies, MVr has also a statistically significant lower hazard ratio in terms of long-term follow-up mortality with RR [0.73; 95% CI, 0.63–0.85; *p* < 0.0001] **(**Fig. 2**)** with moderate heterogeneity observed [*p* = 0.11, I^2^ = 34%] which was solved by removing Brescia 2021 to [*p* = 0.39, I^2^ = 5%] and RR [0.70; 95%CI, 0.60–0.80, *p* < 0.00001].

**Fig. 3:**
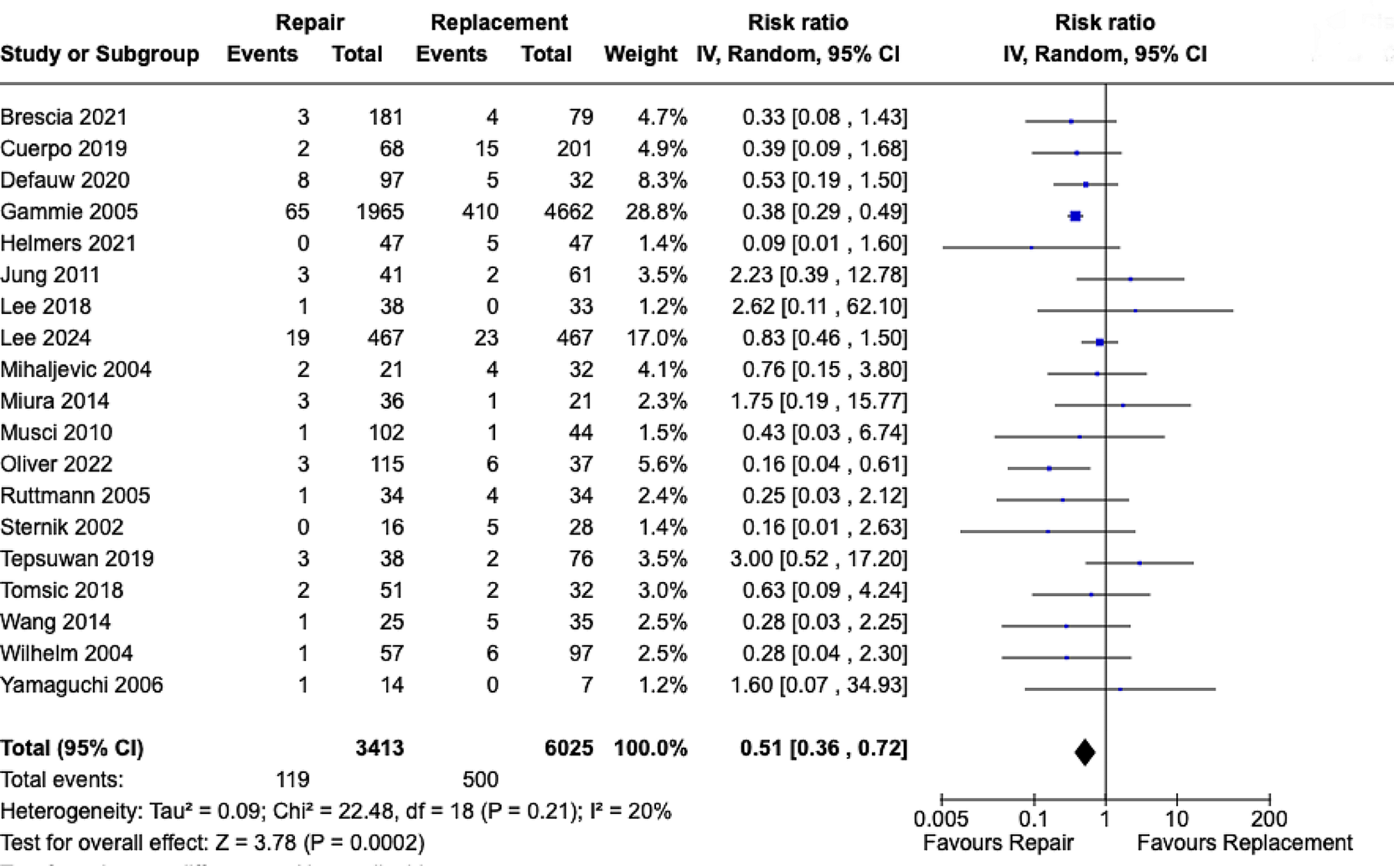
Forrest plot analysis of infective endocarditis recurrence

**Fig. 4:**
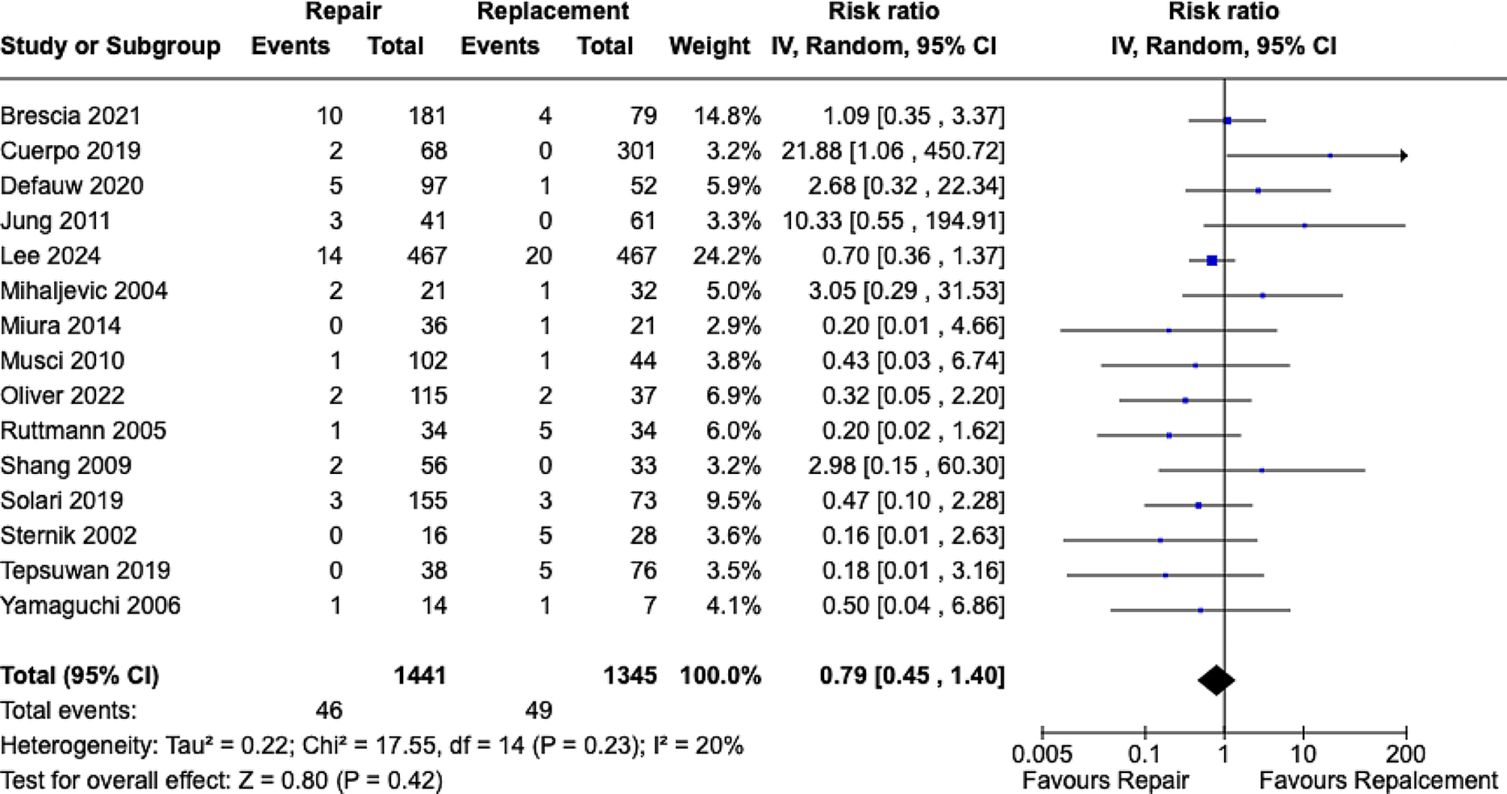
Forrest plot analysis of reoperation

A total of 19 studies were assessed for the risk of IE recurrence with 3413 and 6025.

patients undergoing MVr and MVR respectively. Results showed MVr was associated with a statistically significant lower risk of IE recurrence when compared to MVR with RR [0.51; 95% CI, 0.36–0.72; *p* = 0.0002] **(**Fig. 3**)** with low heterogeneity observed [*p* = 0.21, I^2^ = 20%]. We analyzed 15 studies for assessing the rate of reoperations with 1441 patients undergoing MVr and 1345 undergoing MVR and established that there were no statistically significant differences between both groups with RR [0.79; 95% CI, 0.45–1.40; *p* = 0.42] **(**Fig. 4**)** with low heterogeneity observed [*p* = 0.23, I^2^ = 20%].

Moreover, we performed a meta-regression based on our baseline variables to further add to the robustness of our analysis and deal with any inter-group heterogeneity. In our meta-regression, LVEF showed significant affection of most of our outcomes, besides age which was significantly affecting IE recurrence and reoperation. Further details about our meta-regression can be found in Supplementary Table 3.

## Discussion

To the best of our knowledge, this comprehensive meta-analysis addressing this topic with 23 included studies with 11,802 patients, 7,848 patients in MVR and 3,954 patients in MVr. We found that opting for MVr over MVR entails significantly lower in-hospital mortality rates, higher long-term survival rates, lower risk of IE recurrence, and no difference in reoperation rates. Our findings support the results published earlier by Kang He MS et al. 2022 [2]; however, we reported an additional statistically significant lower rate of IE recurrence after MVr and lesser heterogeneity.

Although MVr’s benefit in patients with IE has been previously established and included in recent guidelines, only 20% of cases are managed by repair [30, 31]. The decision to opt for MVr or MVR is influenced by preoperative patients’ conditions, intraoperative findings, and technical complexity, particularly the surgeon’s qualitative assessment of valvular damage [32, 33]. Other factors such as complex patient presentation, delayed diagnosis, and late surgical referral may also force surgeons to opt for replacing the infected valve. This is the case since the leading indicators of the feasibility of MVr are smaller vegetation dimensions and the absence of severe regurgitation [33, 34]. The choice of which surgery to perform remains multifactorial, and more guidelines now advocate for multidisciplinary decision-making with a specialized “endocarditis team” [35, 36]. It would be useful in this case to analyze certain variables and their eventual influence on the choice of surgery to perform like the preoperative condition of the patient using objective scores, timely nature of surgery; urgent, emergent, or elective, and the microorganism involved. Although mobile vegetation was the indication reported in most of our studies for surgery, current European society of cardiology (ESC) 2023 guidelines states that the most common indication is heat failure [37]. The challenge remains to accurately identify patients where MVr is feasible while outweighing the risks of a failed repair with the benefits of a successful one [32].

Acute bacterial endocarditis was a contraindication for valve repair until about 1990 [37]; however, it is now known that valve repair is not only possible but also safer than valve replacement [30]. The prosthetic mesh could be attributed to most of the feared complications following MVR. MVR not only removes valve tissue that is more resistant to infection than prosthetic mesh, but it also acts as a stimulus for new infection [30]. Even without being infected, mitral valve still has a debate going on between repair and replacement in various scenarios especially after failure of initial repair. In a meta-analysis published by Veerappan et al., there was no difference of whether to re-repair or replacement following failure initial repair in terms of post-operative outcomes [39]. Thus, every modality has its pros and cons making the choice of which is best is solely the surgeons decision based on the patient status as for example the need for anticoagulation following repair increases the incidence of intracranial hemorrhage while the thrombogenic effect of the replaced valve increases the incidence of intracranial embolism [30, 39] thus repair can be contraindicated in patients which a must indication in others. Failure of complete or proper repair has constitute many problems as further surrounding tissue damage or involvement or abscess formation, thus may provide a plausible explanation for why replacement entails a lower risk of IE recurrence and significantly lower in-hospital and long-term mortality rates [40–42]. However, a multi-centric study with more than 10 years follow up showed unfavorable long term outcomes for mitral valve replacement with significant higher risk of mortality and major bleeding in patients with infective endocarditis [43].

On the other hand, the current ESC 2023 guidelines reported directly that “it cannot be concluded that mitral valve repair is superior to replacement due to the high probability of selection patients bias. Valve preservation in acute IE should only be attempted if a durable repair is anticipated and complete eradication of infected tissue can be achieved” [37] which further emphasize that repair is still a very valid and potential option for IE patients when performed in the suitable time, as interventional time has been shown to be a cornerstone in the outcome of these patients as delaying surgery may rise questions and carries hemodynamic instability risks. In a study conducted by Kang et al [44], showed that the earlier the repair the better the outcome of IE patients, yet the patients don’t receive earlier treatment which was evident in the results presented by Gammie et al as only 10–20% of acute cases of IE underwent repair with still showing promising results favoring mitral valve repair [28].

Further patient subgroup analysis by comparing MVr and MVR outcomes in patients with different comorbidities, age groups, or disease severity could provide valuable insight into which patient populations may benefit more from either surgical approach. Additionally, including patient-reported outcomes such as quality of life, satisfaction with surgical outcomes, and impact on daily activities could help understand the patient’s experiences and preferences regarding both surgical approaches. Our results also encourage the need to understand why MVr rates remain low and suggest possible solutions in order to increase its adoption. This could be done by comparing surgical data in different centers, taking into account the number of MVr and MVR surgeries performed, surgeon expertise, and hospital resources. This also introduces the limitation of how factors associated with the choice of MVr or MVR could have biased the comparative results associated with each procedure given that all studies included were observational studies. A pooled analysis would also minimize selection bias however controlling for confounding factors through randomized controlled trials would be optimum. Finally, in addition to MVr’s established clinical benefits, we must also consider its economic implications. A cost-effectiveness analysis could compare the results of both MVr and MVR including factors like follow-up care, rehabilitation, and any necessary reoperations. This could provide insight into improving outcomes and taking advantage of MVr’s benefits in different centers.

### Limitations

It is important to note that the included studies in this meta-analysis had large variabilities in terms of sample size, patient baseline characteristics, disease severity, surgeons’ expertise, and centers’ volume. This is due to the fact that all the included studies are observational studies beside the younger nature of patients undergoing repair might attributed to its favorable ouctomes. Moreover, we couldn’t access individual patients data from the included studies and this may attributed to our high heterogeneity observed beside the difference in baseline characteristics, yet we tried to solve them by sensitivity analysis once found to ensure robustness of our presented results. Thus, the limitations of this study call for further randomized control trials or matching observational studies to be conducted to address the differences in safety and efficacy between MVr and MVR in IE.

## Conclusions

Our results suggest that MVr showed superiority in terms of in-hospital mortality and long-term survival. Furthermore, it has a lower risk of recurrence and valve reoperation. Therefore, MVr is a potentially effective treatment choice that could be considered in most IE patients whenever possible.

### Electronic supplementary material

Below is the link to the electronic supplementary material.

Supplementary Material 1

## Data Availability

All data are available and attached.

## Abbreviations

MVr: Mitral valve repair
MVR: Mitral valve replacement
IE: Infective endocarditis

## References

[1] Chu VH, Park LP, Athan E (2015). Association between surgical indications, operative risk, and clinical outcome in infective endocarditis: a prospective study from the International collaboration on Endocarditis. Circulation.

[2] He K, Song J, Luo H, Su H, Liang W, Bian L (2022). Valve replacement or repair in native mitral valve infective endocarditis—which is better? A meta-analysis and systematic review. J Card Surg.

[3] Page MJ, McKenzie JE, Bossuyt PM (2021). The PRISMA 2020 statement: an updated guideline for reporting systematic reviews. BMJ.

[4] Brooke BS, Schwartz TA, Pawlik TM (2021). MOOSE Reporting guidelines for Meta-analyses of Observational studies. JAMA Surg.

[5] Sterne JAC, Hernán MA, Reeves BC (2016). ROBINS-I: a tool for assessing risk of bias in non-randomized studies of interventions. BMJ.

[6] R Foundation for Statistical Computing, Vienna AR, Core Team R. A Language and Environment for Statistical Computing [Internet]. 2020. https://www.r-project.org/.

[7] Review. Manager (RevMan) [Computer program]. Version 5.4. The Cochrane Collaboration; 2020.

[8] Sternik L, Zehr KJ, Orszulak TA, Mullany CJ, Daly RC, Schaff HV (2002). The advantage of repair of mitral valve in acute endocarditis. J Heart Valve Dis.

[9] Mihaljevic T, Paul S, Leacche M (2004). Tailored surgical therapy for acute native mitral valve endocarditis. J Heart Valve Dis.

[10] Wilhelm MJ, Tavakoli R, Schneeberger K (2004). Surgical treatment of infective mitral valve endocarditis. J Heart Valve Dis.

[11] Ruttmann E, Legit C, Poelzl G (2005). Mitral valve repair provides improved outcome over replacement in active infective endocarditis. J Thorac Cardiovasc Surg.

[12] Shang E, Forrest GN, Chizmar T (2009). Mitral valve infective endocarditis: benefit of early operation and aggressive use of repair. Ann Thorac Surg.

[13] Jung SH, Je HG, Choo SJ, Song H, Chung CH, Lee JW (2011). Surgical results of active infective native mitral valve endocarditis: repair versus replacement. Eur J Cardiothorac Surg.

[14] Miura T, Hamawaki M, Hazama S et al. Outcome of surgical management for active mitral native valve infective endocarditis: a 62:488–98. 10.1007/s11748-014-0377-y 24522750

[15] Wang TKM, Oh T, Voss J, Gamble G, Kang N, Pemberton J (2014). Valvular repair or replacement for mitral endocarditis: 7-year cohort study. Asian Cardiovasc Thorac Ann.

[16] Lee HA, Lin CY, Chen YC (2018). Surgical interventions of isolated active mitral valve endocarditis: predisposing factors and impact of neurological insults on final outcome. Medicine.

[17] Tomšič A, Versteegh MIM, Ajmone Marsan N, Van Brakel TJ, Klautz RJM, Palmen M (2018). Early and late results of surgical treatment for isolated active native mitral valve infective endocarditis. Interact Cardiovasc Thorac Surg.

[18] Cuerpo GP, Valerio M, Pedraz A (2019). Mitral valve repair in infective endocarditis is not inferior to valve replacement: results from a Spanish nationwide prospective registry. Gen Thorac Cardiovasc Surg.

[19] Solari S, De Kerchove L, Tamer S (2019). Active infective mitral valve endocarditis: is a repair-oriented surgery safe and durable?. Eur J Cardiothorac Surg.

[20] Tepsuwan T, Rimsukcharoenchai C, Tantraworasin A (2019). Comparison between mitral valve repair and replacement in active infective endocarditis. Gen Thorac Cardiovasc Surg.

[21] Defauw RJ, Tomsic A, van Brakel TJ, Marsan NA, Klautz RJM, Palmen M (2020). A structured approach to native mitral valve infective endocarditis: is repair better than replacement?. Eur J Cardiothorac Surg.

[22] Scheggi V, Olivotto I, Del Pace S (2020). Feasibility and outcome of mitral valve repair in patients with infective endocarditis. Cardiothorac Surg.

[23] Helmers MR, Fowler C, Kim ST et al. Repair of isolated native mitral valve endocarditis: a propensity matched study. Semin Thorac Cardiovasc Surg. 2021. S1043-0679(21)00283‐5. 10.1053/j.semtcvs.2021.05.025 34197918

[24] Toyoda N, Itagaki S, Egorova NN (2017). Real-world outcomes of surgery for native mitral valve endocarditis. J Thorac Cardiovasc Surg.

[25] Yamaguchi H, Eishi K, Yamachika S, Hisata Y, Tanigawa K, Izumi K, Matsukuma S, Onohara D, Matsumaru I. Mitral valve repair in patients with infective endocarditis. Circ J. 2006;70(2):179 – 83. 10.1253/circj.70.179. PMID: 16434812. 10.1253/circj.70.179 16434812

[26] Brescia AA, Watt TMF, Rosenbloom LM, Williams AM, Bolling SF, Romano MA. Patient and surgeon predictors of mitral and tricuspid valve repair for infective endocarditis. Semin Thorac Cardiovasc Surg. 2022 Spring;34(1):67–77. 10.1053/j.semtcvs.2021.03.017. Epub 2021 Apr 15. PMID: 33865973. 10.1053/j.semtcvs.2021.03.017 33865973

[27] Musci M, Siniawski H, Pasic M, Grauhan O, Weng Y, Meyer R, Yankah CA, Hetzer R. Surgical treatment of right-sided active infective endocarditis with or without involvement of the left heart: 20-year single center experience. Eur J Cardiothorac Surg. 2007;32(1):118 – 25. doi: 10.1016/j.ejcts.2007.02.034. Epub 2007 Apr 6. PMID: 17412606. 10.1016/j.ejcts.2007.02.034 17412606

[28] Gammie JS, O’Brien SM, Griffith BP, Peterson ED. Surgical treatment of mitral valve endocarditis in North America. Ann Thorac Surg. 2005;80(6):2199 – 204. 10.1016/j.athoracsur.2005.05.036. PMID: 16305871. 10.1016/j.athoracsur.2005.05.036 16305871

[29] Oliver L, Leauthier M, Jamme M, Arregle F, Martel H, Philip M, Gouriet F, Casalta JP, Torras O, Casalta AC, Camoin-Jau L, Lavagna F, Renard S, Ambrosi P, Lepidi H, Collart F, Hubert S, Drancourt M, Raoult D, Riberi A, Habib G (2022). Mitral valve repair is better than mitral valve replacement in native mitral valve endocarditis: results from a prospective matched cohort. Arch Cardiovasc Dis.

[30] Lee HA, Chang FC, Yeh JK, Tung YC, Wu VC, Hsieh MJ, Chen CY, Yeh CH, Chu PH, Chen SW. Mitral Valve Repair vs. Replacement by Different Etiologies - A Nationwide Population-Based Cohort Study. Circ J. 2024 Jan 27. 10.1253/circj.CJ-23-0640. Epub ahead of print. PMID: 38281764. 10.1253/circj.CJ-23-0640 38281764

[31] Pettersson GB, Hussain ST (2019). Current AATS guidelines on surgical treatment of infective endocarditis. Ann Cardiothorac Surg.

[32] Scheggi V, Olivotto I, Del Pace S, Zoppetti N, Brunetto A, Marchionni N (2020). Feasibility and outcome of mitral valve repair in patients with infective endocarditis. Cardiothorac Surg.

[33] Nappi F, Spadaccio C, Dreyfus J, Attias D, Acar C, Bando K (2018). Mitral endocarditis: a new management framework. J Thorac Cardiovasc Surg.

[34] Cahill TJ, Prendergast BD (2016). Infective endocarditis. Lancet.

[35] Baddour LM, Wilson WR, Bayer AS, Fowler VG, Tleyjeh IM, Rybak MJ (2015). Infective endocarditis in adults: diagnosis, antimicrobial therapy, and management of complications: A Scientific Statement for Healthcare professionals from the American Heart Association. Circulation.

[36] Habib G, Lancellotti P, Antunes MJ, Bongiorni MG, Casalta JP, Del Zotti F (2015). 2015 ESC guidelines for the management of infective endocarditis: the Task Force for the management of infective endocarditis of the European Society of Cardiology (ESC)endorsed by: European Association for Cardio-thoracic surgery (EACTS), the European Association of Nuclear Medicine (EANM). Eur Heart J.

[37] Delgado V, Ajmone Marsan N, de Waha S, Bonaros N, Brida M, Burri H, Caselli S, Doenst T, Ederhy S, Erba PA, Foldager D, Fosbøl EL, Kovac J, Mestres CA, Miller OI, Miro JM, Pazdernik M, Pizzi MN, Quintana E, Rasmussen TB, Ristić AD, Rodés-Cabau J, Sionis A, Zühlke LJ, Borger MA, ESC Scientific Document Group. 2023 ESC Guidelines for the management of endocarditis. Eur Heart J. 2023;44(39):3948–4042. 10.1093/eurheartj/ehad193. Erratum in: Eur Heart J. 2023 Sep 20;: Erratum in: Eur Heart J. 2024 Jan 1;45(1):56. PMID: 37622656.

[38] Dreyfus G, Serraf A, Jebara VA, Deloche A, Chauvaud S, Couetil JP (1990). Valve repair in acute endocarditis. Annals Thorac Surg.

[39] Veerappan M, Cheekoty P, Sazzad F, Kofidis T (2020). Mitral valve re-repair vs replacement following failed initial repair: a systematic review and meta-analysis. J Cardiothorac Surg.

[40] Fathy MH. and others, Infective endocarditis of mitral valve, valve replacement or repair?- a meta-analysis of 10,965 patients, European Heart Journal, Volume 43, Issue Supplement_2, October 2022, ehac544.2122, 10.1093/eurheartj/ehac544.2122.

[41] Martínez-Sellés M, Muñoz P, Arnáiz A, Moreno M, Gálvez J, Rodríguez-Roda J, de Alarcón A, García Cabrera E, Fariñas MC, Miró JM, Montejo M, Moreno A, Ruiz-Morales J, Goenaga MA, Bouza E (2014). Spanish collaboration on Endocarditis — Grupo De Apoyo Al Manejo De La Endocarditis infecciosa en ESpaña (GAMES). Valve surgery in active infective endocarditis: a simple score to predict in-hospital prognosis. Int J Cardiol.

[42] Chirillo F, Scotton P, Rocco F, Rigoli R, Borsatto F, Pedrocco A, De Leo A, Minniti G, Polesel E, Olivari Z. Impact of a multidisciplinary management strategy on the outcome of patients with native valve infective endocarditis. Am J Cardiol. 2013;112(8):1171-6. doi: 10.1016/j.amjcard.2013.05.060. Epub 2013 Jul 5. PMID: 23831163. 10.1016/j.amjcard.2013.05.060 23831163

[43] Anttila V, Malmberg M, Gunn J, Rautava P, Kytö V (2021). Infective endocarditis and outcomes of mitral valve replacement. Eur J Clin Invest.

[44] Kang DH, Kim YJ, Kim SH, Sun BJ, Kim DH, Yun SC, Song JM, Choo SJ, Chung CH, Song JK, Lee JW, Sohn DW. Early surgery versus conventional treatment for infective endocarditis. N Engl J Med. 2012;366(26):2466-73. 10.1056/NEJMoa1112843. PMID: 22738096. 10.1056/NEJMoa1112843 22738096

